# GRK2 knockdown in mice exacerbates kidney injury and alters renal mechanisms of blood pressure regulation

**DOI:** 10.1038/s41598-018-29876-8

**Published:** 2018-07-30

**Authors:** Elena Tutunea-Fatan, Khaled S. Abd-Elrahman, Jean-Francois Thibodeau, Chet E. Holterman, Brian J. Holleran, Richard Leduc, Christopher R. J. Kennedy, Robert Gros, Stephen S. G. Ferguson

**Affiliations:** 1 Vascular Biology Group, Robarts Research Institute, University of Western Ontario, London, Ontario N6A 5K8 Canada; 2 Department of Physiology and Pharmacology, University of Western Ontario, London, Ontario N6A 5K8 Canada; 3 University of Ottawa Brain and Mind Research Institute and Department of Cellular and Molecular Medicine, University of Ottawa, Ottawa, Ontario K1H 8M5 Canada; 4 Kidney Research Center, Ottawa Hospital Research Institute, Ottawa, Ontario K1H 8M5 Canada; 5 Department of Pharmacology and Toxicology, Faculty of Pharmacy, Alexandria University, Alexandria, 21521 Egypt; 6 Department of Pharmacology-Physiology, Faculty of Medicine and Health Sciences, Université de Sherbrooke, Sherbrooke, Quebec J1H 5N4 Canada

## Abstract

The renin-angiotensin system regulates blood pressure and fluid balance in the body primarily via angiotensin receptor 1 (AT1R). Renal AT1R was found to be primarily responsible for Ang II-mediated hypertension. G protein-coupled receptor kinase 2 (GRK2) modulates AT1R desensitization and increased GRK2 protein expression is reported in hypertensive patients. However, the consequences of GRK2 inhibition on kidney functions remain unknown. We employed shGRK2 knockdown mice (shGRK2 mice) to test the role of GRK2 in kidney development and function that can be ultimately linked to the hypertensive phenotype detected in shGRK2 mice. GRK2 knockdown reduced kidney size, nephrogenesis and glomerular count, and impaired glomerular filtration. Glomerular damage in adult shGRK2 mice was associated with increased renin- and AT1R-mediated production of reactive oxygen species. The AT1R blocker, Losartan, normalized elevated blood pressure and markedly improved glomerular filtration in the shGRK2 knockdown mice. Our findings provide evidence for the crucial role of GRK2 in renal regulation of blood pressure. It also suggests that the detrimental outcomes of GRK2 inhibitors on the kidney should be carefully examined when used as antihypertensive.

## Introduction

The renin-angiotensin system (RAS) plays a crucial role in the regulation of blood pressure, body fluid and electrolyte balance through the main effector peptide angiotensin II (Ang II). Ang II exerts these physiological actions in many target organs in the cardiovascular system including the kidneys, heart and blood vessels^1,2^. The majority of known biological actions of Ang II are mediated through the G-protein coupled receptor (GPCR), Angiotensin receptor 1 (AT1R)^3^. Renal AT1R modulates a multitude of functions in the kidney and is expressed in renal vasculature, glomeruli and proximal tubules and is believed to be primarily responsible for Ang II-mediated hypertension^4,5^. Ang II via AT1R has direct effects on afferent and efferent arterioles causing vasoconstriction that results in reduction of renal blood flow and glomerular filtration rate (GFR). Moreover, AT1R activation stimulates secretion of aldosterone from the adrenal cortex, which promotes sodium and water reabsorption by the distal tubule^2,3^. Non-hemodynamic functions of Ang II in the kidney include acting as a potent proinflammatory, stimulating reactive oxygen species (ROS) production, inducing both hypertrophy and proliferation of mesangial cells, and profibrogenic actions (stimulation of matrix synthesis and inhibition of matrix degradation)^4,6^.

As a GPCR, AT1R activity is controlled at a post-translational level to achieve a precise balance between molecular mechanisms regulating receptor signaling and desensitization. G protein-coupled receptor kinase (GRK) dictates GPCR desensitization by phosphorylating the carboxy terminal tail and third intracellular loop of the receptor and uncoupling the activated GPCRs from their downstream signaling effectors^7,8^. In addition, GRKs regulate cellular responses in a phosphorylation-independent manner due to their ability to interact with several proteins involved in trafficking and signaling^9,10^. Thus, alterations in GRK activity and/or expression in the kidney is expected to have an impact on AT1R signaling and could be associated with pathophysiological consequences.

Among the seven mammalian GRKs, GRK2 was the only isoform that caused embryonic lethality after homozygous deletion in mice suggesting that it is the most crucial GRK isoform^11^. Specifically, GRK2 is unique with respect to other GRK family members since it mediates the RGS homology domain-independent desensitization of only G_αq/11_-coupled GPCRs including AT1R^12^. Human studies detected an increase GRK2 protein expression in lymphocytes from hypertensive and heart failure patients presumably due to the desensitization of β adrenergic receptors (βARs) that mediate vasodilatation^13,14^. These findings suggested that inhibition of GRK2 could represent a viable therapeutic strategy for the treatment of hypertension and heart failure^15,16^. Given the crucial role of AT1R in multitude of physiological actions, previous studies suggested that while partial inhibition of GRK2 might be beneficial^17,18^, total inhibition of its expression may cause alterations in AT1R signaling that could be associated with pathophysiological consequences in the cardiovascular system. Indeed, global knockdown of GRK2 using small hairpin interfering RNA (shRNA) resulted in mice that show unopposed AT1R signaling leading to increased vasoconstrictor activity and hypertension^19^. Although RAS and AT1R play an essential role in renal physiology and blood pressure homeostasis, the consequences of complete GRK2 inhibition on kidney function and development remains largely unclear.

Here, we utilized shGRK2 knockdown mice (shGRK2 mice) to explore the role of GRK2 in kidney function and development. shGRK2 mice present with reduced kidney size and impaired electrolyte balance. GRK2 knockdown altered nephrogenesis and was associated with reduced glomerular count and filtering capacity in adult mice. shGRK2 mice were spontaneously hypertensive and the AT1R blocker, Losartan, reduced blood pressure and improved GFR. Interestingly, GRK2 knockdown did not alter AT1R membrane density but enhanced renin- and AT1R-mediated signaling, specifically increased ROS production that contributed to glomerular damage. Together, our data provide evidence for the crucial role of GRK2 in renal mechanisms of blood pressure homeostasis and indicate that total inhibition of GRK2 can have deleterious outcomes on kidney function and development.

## Results

### GRK2 knockdown alters kidney morphometry and filtering function

**Figure 1:**
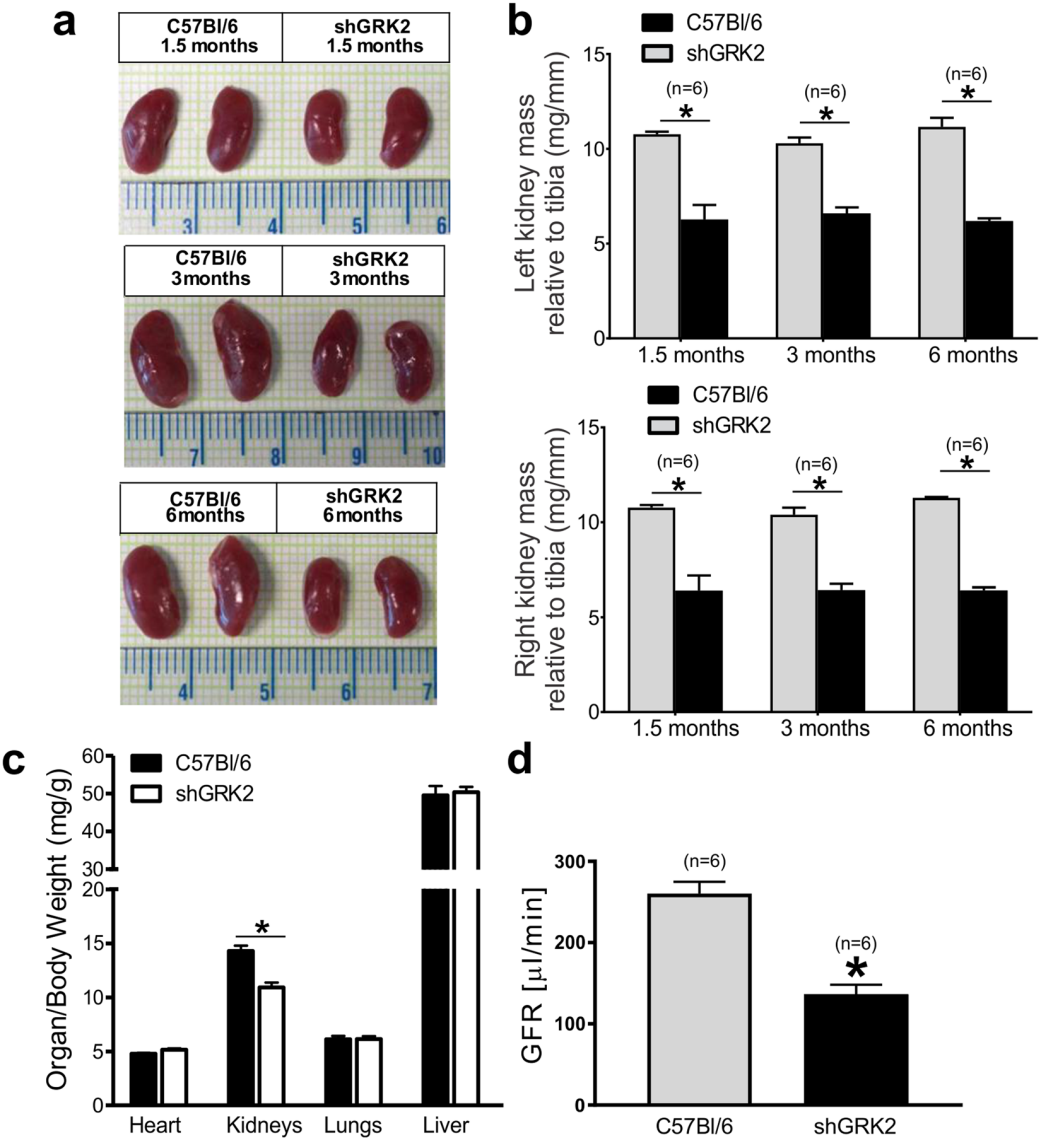
GRK2 knockdown alters kidney morphometry and reduces glomerular filtration rate (GFR). (**a**) Representative images of right and left kidneys from 1.5-, 3- and 6-month-old shGRK2 and age-matched C57Bl/6 mice. (**b**) Mean ± S.E.M of kidney mass (right & left) relative to tibia length of shGRK2 and C57Bl/6 mice at 1.5, 3 and 6 months of age (n = 6). (**c**) Mean ± S.E.M of organ mass (heart, lungs, kidneys, and liver) relative to body weight in 3-month-old shGRK2 and age-matched C57Bl/6 mice (n = 6). (**d**) Mean ± S.E.M of glomerular filtration rate (GFR) measured by FITC-inulin clearance in 6-month-old shGRK2 and age-matched control C57Bl/6 mice (n = 6). * denotes significantly different at (p < 0.05, unpaired Student’s t test).

In collaboration with the late Dr. Hubert H. M. Van Tol, we employed shRNA using a U6 mouse polymerase III promoter as a transgene to universally knockdown, but not knockout, GRK2 protein expression in transgenic mice^19^. Male shGRK2 transgenic mice were significantly smaller in size with lower body weight compared to wild-type littermates^19^. We assessed the organ-to-body weight ratios for major internal organs (hearts, lungs, and livers) at different stages of adulthood (1.5, 3 and 6 months) and detected no significant differences between shGRK2 and age-matched C57B6/l mice (Fig. 1a). Interestingly, we observed a significant reduction in kidney/body weight and kidney mass/tibia length of shGRK2 mice compared to age-matched controls at all ages studied (Fig. 1b and c). To assess whether renal hypotrophy had an impact on renal function, we assessed GFR by measuring FITC-inulin clearance in 6-month-old shGRK2 and control mice. GRK2 knockdown was associated a significant GFR reduction compared to control mice (Fig. 1d). Moreover, urinary electrolyte analysis of 1, 3 and 6-month-old shGRK2 mice revealed reductions in Na^+^ content compared to age-matched controls (Table 1). We also detected an increase in serum potassium and blood urea nitrogen in 6-month-old shGRK2 compared to age-matched controls consistent with advanced renal injury associated with GFR reduction (Table 2). It is worth noting that we observed a reduction in serum creatinine in 1 and 3-month-old shGRK2 mice. Since serum creatinine can serve as surrogate maker for muscle mass^20^, this decrease in serum creatinine could be attributed to reduced body weight and muscle mass following GRK2 knockdown^19^ (Table 2). The these findings indicate that GRK2 genetic knockdown has kidney-specific detrimental effects on development and morphology that translate into impaired filtering capacity and electrolyte handling later in adult mice.

**Table 1:** Biochemical analysis of urine from shGRK2 mice.

Analyte (unit)	1-month old (n = 6)		3-month-old (n = 6)		6-month-old (n = 6)	
	C57Bl/6	shGRK2	C57Bl/6	shGRK2	C57Bl/6	shGRK2
Sodium (mmol/L)	199 ± 46	**149** **±** 44*	160 ± 61	**112** **±** 23*	175 ± 30	**105** **±** 53*
Potassium (mmol/L)	72 ± 23	87 ± 4	91 ± 6	104 ± 14	94 ± 6	83 ± 14
Chloride (mmol/L)	198 ± 60	232 ± 61	232 ± 43	245 ± 56	233 ± 31	165 ± 66
Calcium (mg/dL)	5.2 ± 1.2	4.9 ± 1.3	5.2 ± 1.3	6.6 ± 1.4	5.1 ± 0.2	5.9 ± 0.01
Albumin (g/L)	0.15 ± 0.1	0.1 ± 0.01	0.7 ± 0	0.5 ± 0.34	0.6 ± 0.1	0.7 ± 0.05
Creatinine (mg/dL)	25 ± 12	26 ± 5	37 ± 4	30 ± 16	38 ± 2	40 ± 1

Biochemical analysis of urine collected from 1-, 3- and 6-month-old shGRK2 and age-matched C57Bl/6 mice (n = 6). * denotes significantly different at (p < 0.05, unpaired Student’s t test).

**Table 2:** Biochemical analysis of serum from shGRK2 mice.

Analyte (unit)	1-month old (n = 6)		3-month-old (n = 6)		6-month-old (n = 6)	
	C57Bl/6	shGRK2	C57Bl/6	shGRK2	C57Bl/6	shGRK2
Sodium (mmol/L)	149 ± 0.4	147 ± 2.1	149 ± 3.2	151 ± 2.0	150 ± 2.8	146 ± 8.9
Potassium (mmol/L)	4.4 ± 0.10	4.1 ± 0.07	4.0 ± 0.32	4.1 ± 0.61	3.9 ± 0.38	**5**.**2** **±** **1**.5*
Chloride (mmol/L)	114 ± 1.2	111 ± 1.6	113 ± 2.8	118 ± 2	113 ± 2.4	113.2 ± 8.4
Calcium (mg/dL)	9.6 ± 0.3	9.9 ± 0.1	8.9 ± 0.3	8.9 ± 0.2	9.1 ± 0.2	9.2 ± 0.4
Albumin (g/L)	26 ± 1.0	26.3 ± 0.7	27.0 ± 1.1	26 ± 1.3	27.0 ± 1.5	25.1 ± 2.3
Blood Urea (mg/dL)	19.1 ± 1.3	22.3 ± 2.0	24.7 ± 3.4	28.2 ± 2.4	22.7 ± 2.9	**28**.**0** **±** **1**.4*
Creatinine (mg/dL)	0.22 ± 0.02	**0**.**18** **±** **0**.01*	0.22 ± 0.01	**0**.**18** **±** **0**.03*	0.19 ± 0.01	0.20 ± 0.01
Glucose (mg/dL)	213 ± 43	252 ± 26	219 ± 38	234 ± 50	270 ± 38	256 ± 45

Biochemical analysis of serum samples collected from 1-, 3- and 6-month-old shGRK2 and age-matched C57Bl/6 mice (n = 6). * denotes significantly different at (p < 0.05, unpaired Student’s t test).

### GRK2 knockdown alters glomerular maturation and number

**Figure 2:**
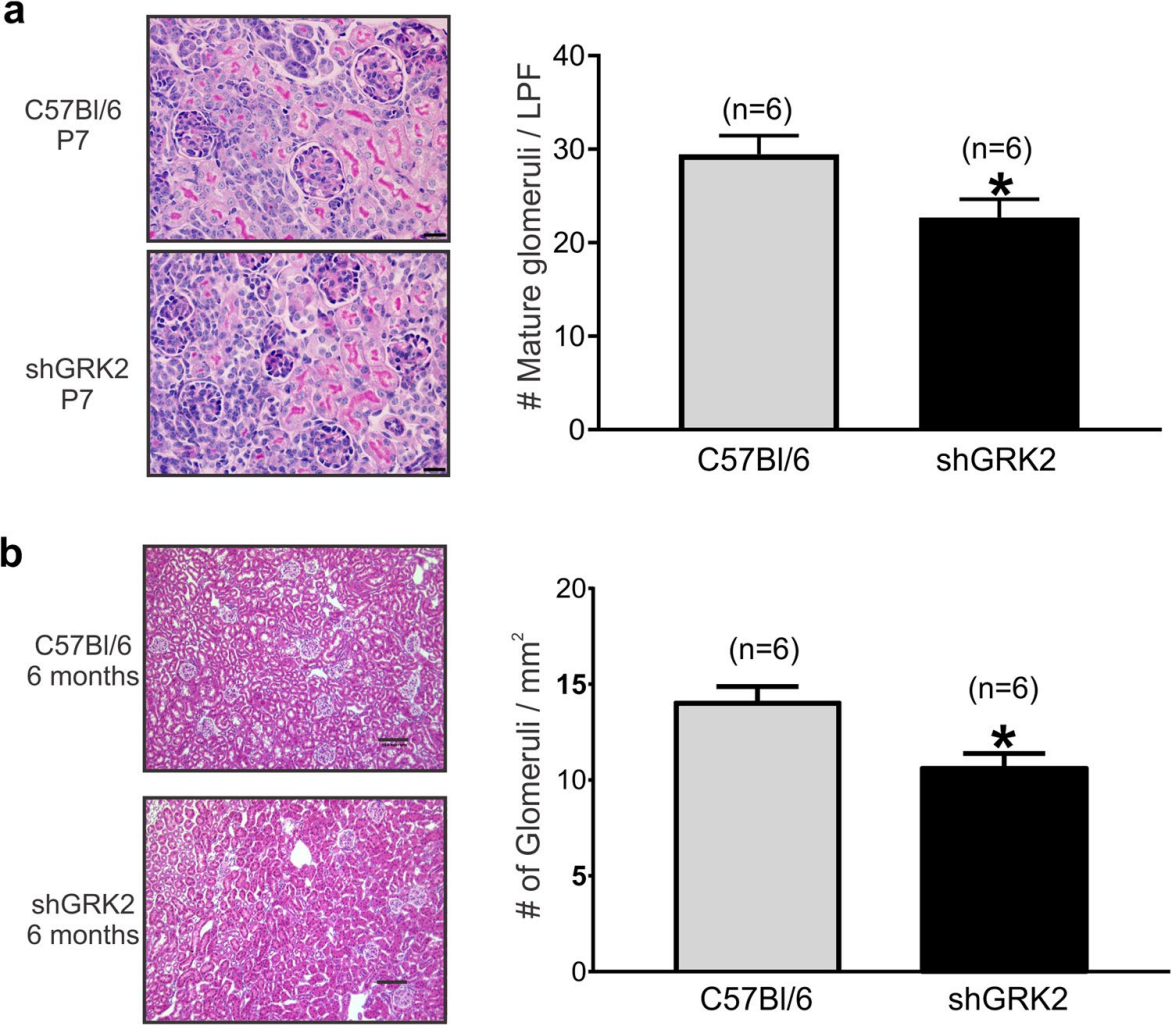
GRK2 knockdown alters glomerular maturation and count. (**a**) Representative images of Periodic-Acid Schiff stained histological kidney sections and mean ± S.E.M of the number of mature glomeruli in low power field (LPF) from postnatal day 7 (P7) shGRK2 and age-matched C57Bl/6 mice. Scale bar is 20 µm (n = 6). (**b**) Representative images of H&E stained histological kidney sections and mean ± S.E.M of the number of mature glomeruli/mm^2^ from 6-month-old shGRK2 and age-matched C57Bl/6 mice. Scale bar is 100 µm (n = 6). * denotes significantly different at (p < 0.05, unpaired Student’s t test).

**Figure 3:**
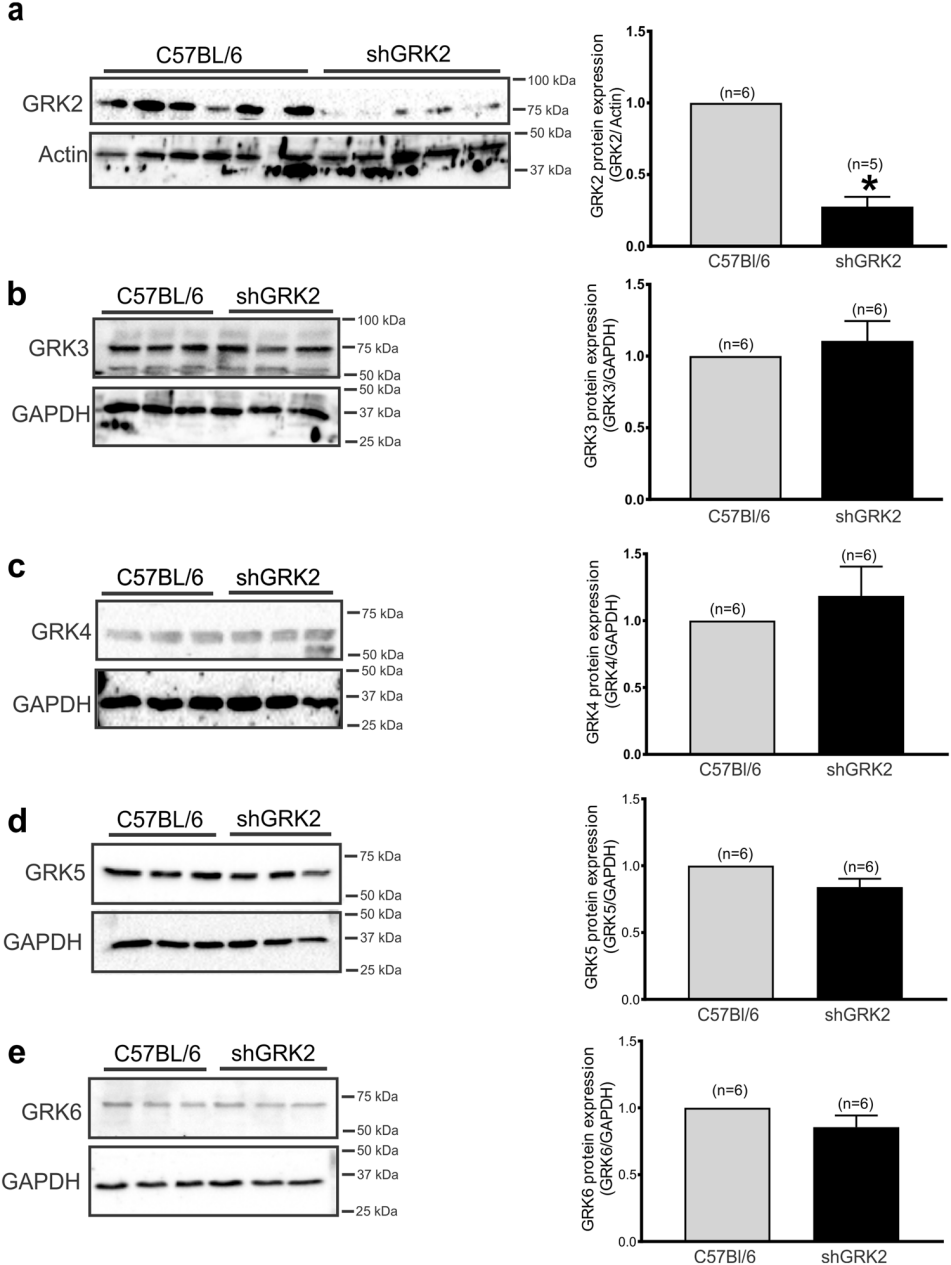
Expression of GRK3, 4, 5 and 6 is not changed in shGRK2 kidneys. Representative western blots and mean ± S.E.M for the densiometric analysis of (**a**) GRK2, (**b**) GRK3, (**c**) GRK4, (**d**) GRK5, and (**e**) GRK6 expression with the corresponding loading control glyceraldehyde 3-phosphate dehydrogenase (GAPDH) or actin in renal cortex of 6-month-old shGKR2 and age-matched C57Bl/6 mice (n = 5–6). GRK band intensity was normalized to corresponding actin (for GRK2) or GAPDH (for GRK3, 4, 5, 6) band and expressed as a fraction of the C57Bl/6 value. * denotes significantly different at (p < 0.05, unpaired Student’s t test).

The reduced kidney size and filtration in shGRK2 mice could be due to altered glomerulogenesis during development that persisted in adult mice. Thus, we isolated kidneys from shGRK and C57Bl/6 mice at postnatal day 7 (P7). Histological examination of H&E-stained kidney sections revealed a lower number of mature glomeruli in shGRK2 mice compared to control (Fig. 2a). This loss in mature glomeruli was not rescued later in life since the reduction in glomerular count persisted in 6-month-old shGRK2 mice (Fig. 2b). There is a possibility that compensatory changes in other GRKs may play a role in the developmental changes that we detected in shGRK2 mice. Thus, we tested kidney samples from 6-month-old shGRK2 mice and although we confirmed GRK2 knockdown, no significant changes in GRK3, 4, 5 and 6 was detected when compared age-matched controls (Fig. 3). Together, our results indicated that the targeted knockdown of GRK2 alters glomeruli formation during development that may underlie the reduced kidney size and function of adult shGRK2 mice.

### GRK2 knockdown increases renal and plasma renin expression

**Figure 4:**
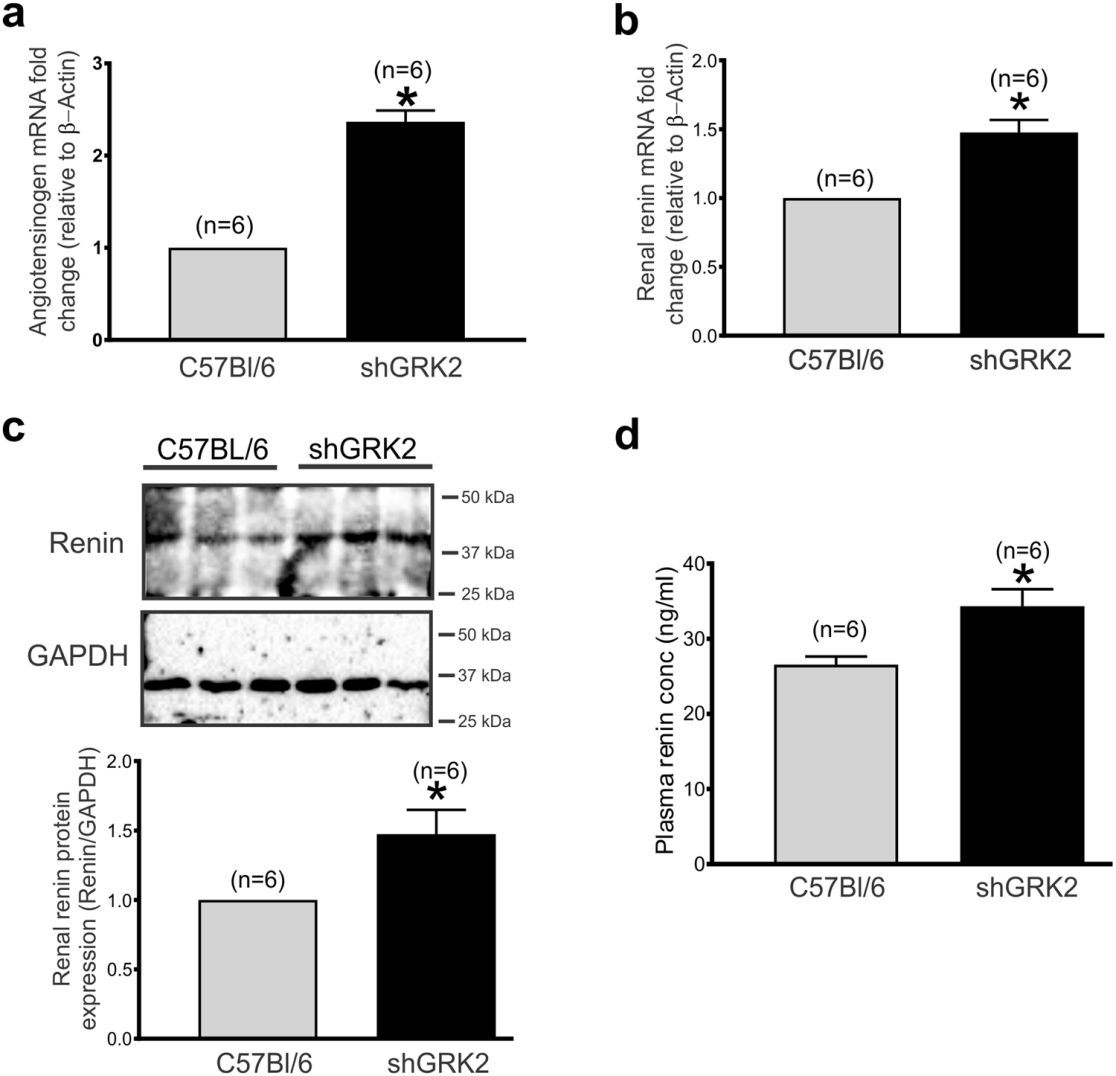
GRK2 knockdown increases renal and plasma renin expression. Mean ± S.E.M showing folds change in (**a**) renal angiotensinogen and (**b**) renin mRNA expression from 6-month-old shGRK2 over age-matched C57Bl/6 mice. Expressions levels are normalized to β-actin (n = 6). (**c**) Representative western blots and mean ± S.E.M for the densiometric analysis of renin protein expression with the corresponding loading control Glyceraldehyde 3-phosphate dehydrogenase (GAPDH) in renal cortex of 6-month-old shGKR2 and age-matched C57Bl/6 mice (n = 6). Renin band intensity was normalized to corresponding GAPDH band intensity and expressed as a fraction of the C57Bl/6 value (n = 6). (**d**) Mean ± S.E.M of plasma renin levels in 6-month-old shGRK2 and age-matched C57Bl/6 mice measured by Mouse renin ELISA kit as per manufactures’ instructions (n = 6). * denotes significantly different at (p < 0.05, unpaired Student’s t test).

Reduction in GFR and renal blood flow stimulate the proteolytic cleavage of the precursor, prorenin, releasing renin directly into the blood stream. Renin then triggers a cascade of events that leads to formation of Ang II and activation of RAS^1,2,6^. We detected an increase in renal mRNA of angiotensinogen (Fig. 4a) and, renal mRNA and protein expression of renin in 6-month-old shGRK2 compared to age-matched controls (Fig. 4b and c). We also detected and increase in plasma renin concentration in shGRK2 mice at 6 months of age (Fig. 4d). Together, these data suggest that altered GFR and renal blood flow as result of impairment in the kidney filtering capacity in shGRK2 can trigger the rate-limiting step in RAS activation potentially leading to augmented renin- and Ang II-mediated responses.

### GRK2 knockdown exacerbates ROS production and glomerular injury

**Figure 5:**
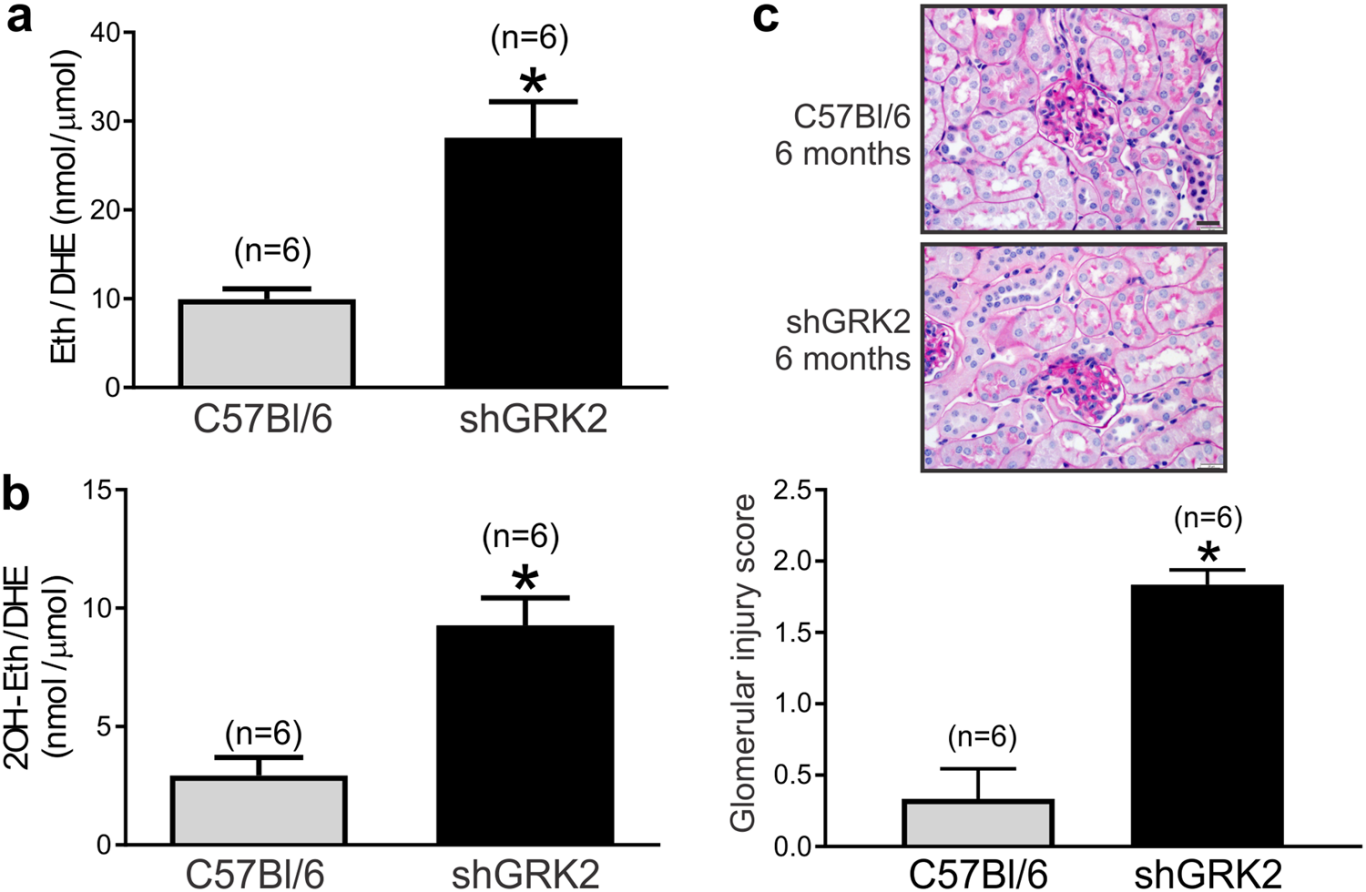
ROS production and glomerular injury are exacerbated in shGRK2 mice. Mean ± S.E.M for renal cortex levels (nmol/µmol) of (**a**) ethidium (Eth) and (**b**) 2-hydroxydihydroethidium (2OH-Eth) after incubation with dihydroethidium (DHE) and separation by high-performance liquid chromatography (HPLC). Eth and 2OH-Eth levels were normalized to dihydroethidium (DHE) levels. (**c**) Representative images of Periodic-Acid Schiff stained glomerulus from 6-month-old shGRK2 and age-matched C57Bl/6.Scale bar is 20 µm. Mean ± S.E.M of the glomerular injury score based on mesangial matrix area done by trained pathologist in blinded manner (n = 6). * denotes significantly different at (p < 0.05, unpaired Student’s t test).

Previous reports showed that Ang II-mediated activation of renal AT1R increases ROS production and extracellular matrix proteins^6,21–23^. Renin, either directly via activating pro-renin receptors or indirectly via increasing Ang II production, can also enhance oxidative stress through production of superoxide (O_2_^−^) and hydrogen peroxide (H_2_O_2_) and increase synthesis of extracellular matrix proteins in renal collecting duct and mesangial cells^24,25^. We tested whether the RAS activation that we detected in shGRK2 kidneys was accompanied by increased renal O_2_^−^ and H_2_O_2_. Levels of oxidation products of DHE, superoxide-dependent 2OH-Eth and H_2_O_2_-dependent Eth, were significantly elevated in renal cortex of 6-month-old shGRK2 compared to age-matched controls (Fig. 5a and b). Periodic Acid-Schiff’s staining revealed that shGRK2 glomerular membranes scored higher for sclerosis compared to controls indicating an increase in extracellular matrix proteins production, specifically collagen (Fig. 5c). These findings suggest that GRK2 knockdown increases production of basal ROS and extracellular matrix proteins, possibly due to RAS activation and enhanced AT1R signaling, that contributed to glomerular injury and impaired renal function of shGRK2 mice.

### GRK2 knockdown does not alter AT1R affinity or density in the kidney

**Figure 6:**
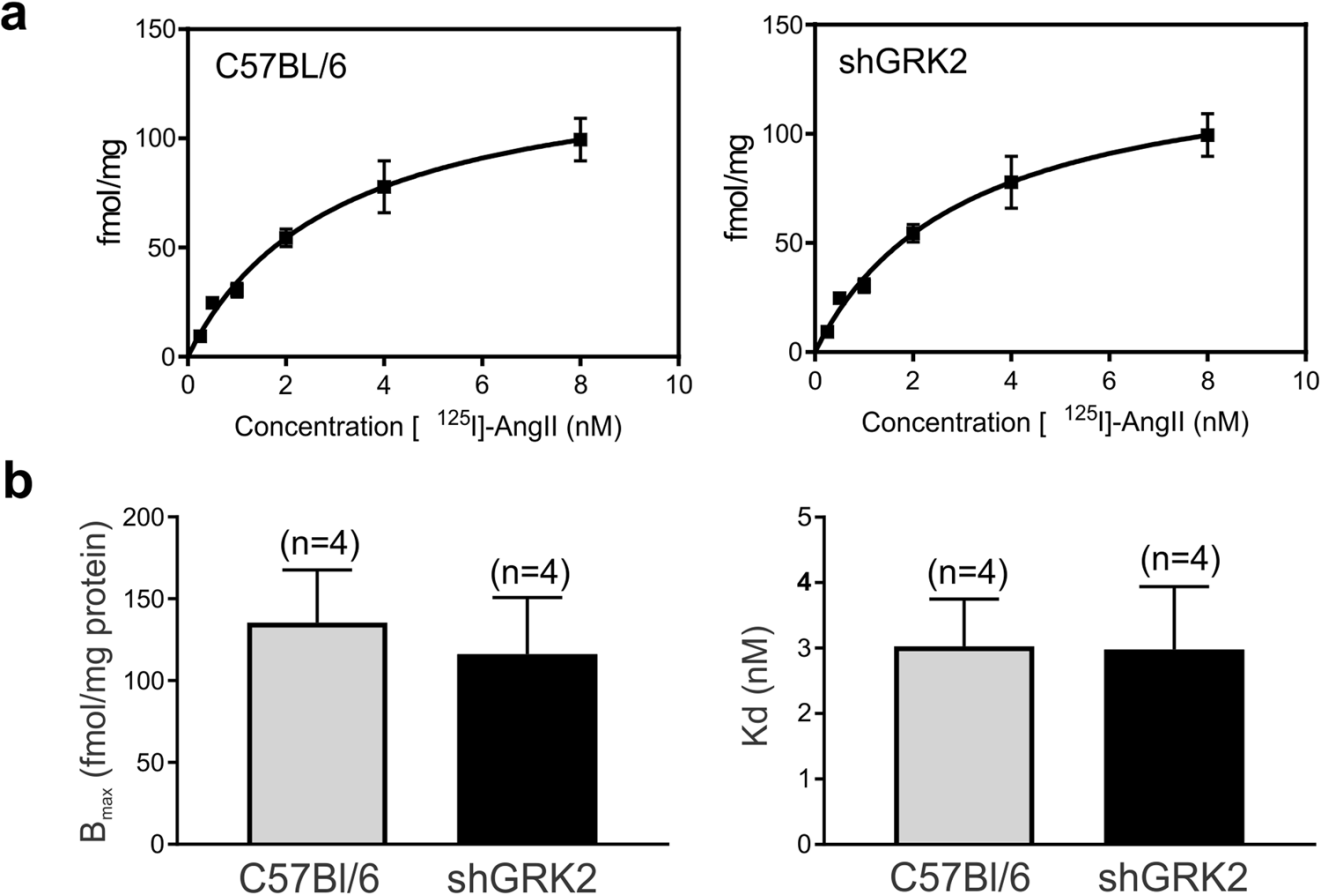
AT1R affinity and density are not changed in shGRK2 kidneys. (**a**) Representative saturation binding curves and (**b**) mean ± S.E.M of binding affinities (K_d_, nM) and maximal binding capacities (B_max_, fmol/mg protein) for [^125^I]-Ang II in renal cortex of 6-month-old shGRK2 mice and age-matched C57Bl/6 mice (n = 4 independent experiments run in duplicates). Lack of significant difference was verified using unpaired Student’s t test.

GRK2-mediated desensitization of G_αq_/11-coupled GPCRs following agonist stimulation triggers a cascade of signaling events that eventually leads to receptor internalization^7,26,27^. We tested whether GRK2 knockdown would reduce AT1R internalization and degradation that could underlie renal dysfunction and hypertensive phenotype in shGRK2 mice. Radioligand binding assay using ^125^I Ang II did not show a significant change in B_max_ and K_d_ between shGRK2 and age-matched C57Bl/6 mice indicating equal AT1R expression between the two mouse lines (Fig. 6a and b). This finding indicates the GRK2 knockdown does not affect renal AT1R density, and the changes in renal function of shGRK2 mice are due altered AT1R signaling rather than expression.

### AT1R blockade in shGRK2 mice rescues the hypertensive phenotype and reduced GFR

**Figure 7:**
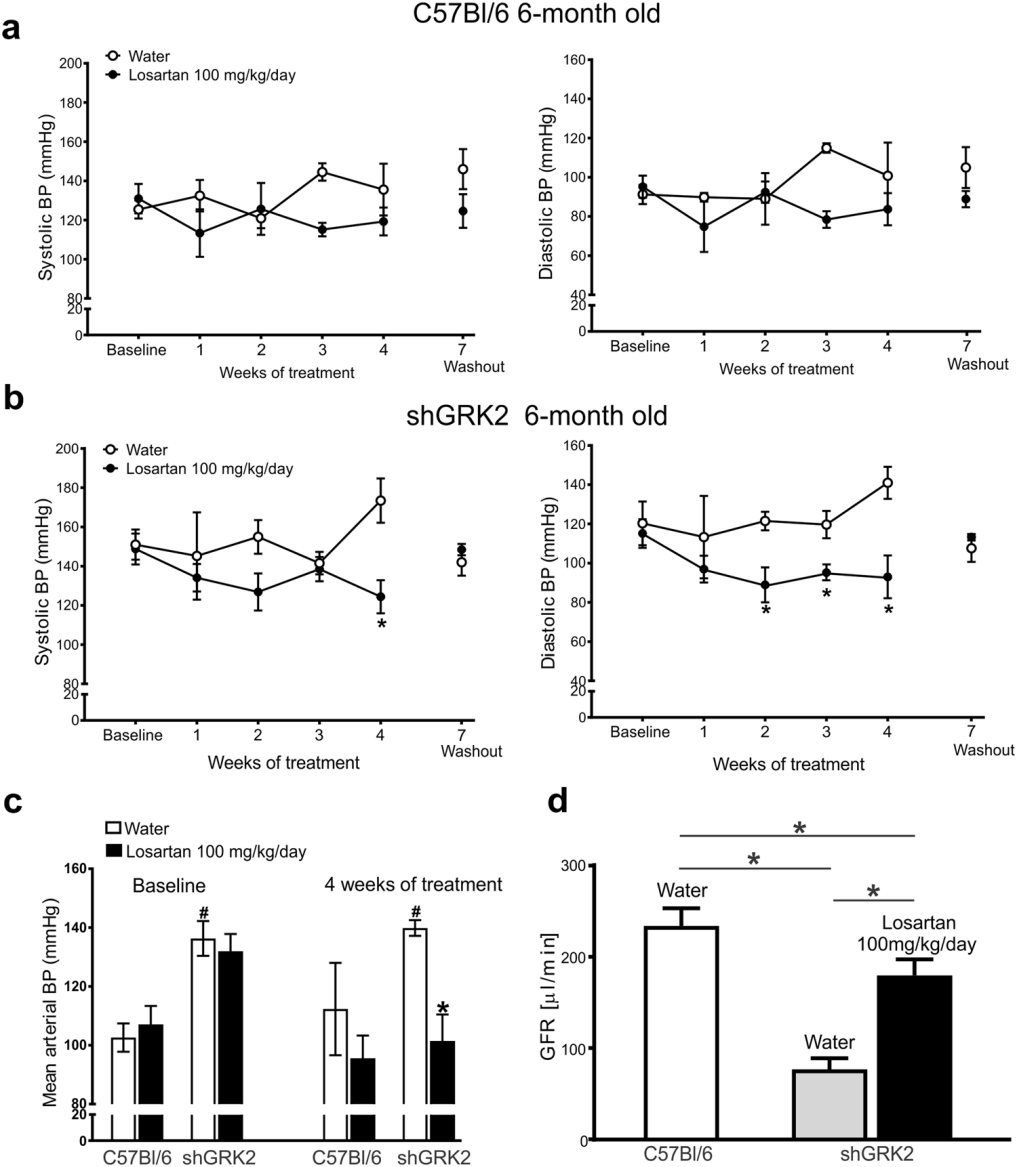
Losartan mitigates the hypertensive phenotype and impaired GFR in shGRK2 mice. Mean ± S.E.M of systolic (left) and diastolic blood pressure (right) from 6-month-old C57Bl/6 (**a**) and age-matched shGRK2 (**b**) using the non-invasive CODA tail-cuff blood pressure system. Blood pressure values were measured before treatment start (baseline), weekly during the 4-week period of treatment (with either plain drinking water or 100 mg/kg/day Losartan in drinking water), and 3 weeks after washout ended (washout) (n = 3–6). * denotes significantly different from corresponding plain water value at (p < 0.05, two-way ANOVA). (**c**) Mean ± S.E.M of mean arterial blood pressure for 6-month-old shGRK2 and age-matched C57Bl/6 mice before (baseline) and 4 weeks after treatment with either plain drinking water or Losartan (n = 3–6). ^#^ denotes significantly different at (p < 0.05, two-way ANOVA) from baseline C57Bl/6 receiving plain water. * denotes significantly different from corresponding plain water value at (p < 0.05, two-way ANOVA). (**d**) Mean ± S.E.M of glomerular filtration rate (GFR) measured by FITC-inulin clearance in 6-month-old shGRK2 and age-matched control C57Bl/6 mice after 4 weeks of treatment with either plain drinking water or Losartan (n = 3–6). * denotes significantly different at (p < 0.05, one-way ANOVA).

The increase in renal renin protein expression and plasma concentration in shGRK2 mice is expected to increase the production of Ang II. The elevated level of Ang II and the enhanced AT1R signaling, due to lack of receptor desensitization, would be associated with elevated blood pressure and constriction of afferent and efferent arterioles resulting in further reduction in GFR and renal blood flow^1,2,6^. Thus, AT1R blockade should be able to ameliorate renal injury and hypertension in shGRK2 mice. To test that we treated 6-month-old shGRK2 mice with the AT1R blocker, Losartan, in drinking water at a dose of 100 mg/kg/day for 4 weeks^28,29^ and measured blood pressure and GFR. No significant change was detected in systolic, diastolic and mean blood pressure in C57Bl/6 mice over the treatment period with losartan compared to water (Fig. 7a and c). By the end of the 4-week treatment period, losartan significantly ameliorated the hypertensive phenotype in shGRK2 mice as demonstrated by a reduction in systolic, diastolic and mean blood pressure (Fig. 7b and c). After a three-week wash out period, blood pressure recovered to pre-treatment values in shGRK2 mice. Moreover, 4-week treatment with losartan rescued the reduction from GFR observed in shGRK2 mice, however GFR values of losartan-treated shGRK2 mice remained significantly lower than healthy age-matched C57Bl/6 mice (Fig. 7d). Together, these findings emphasize that GRK2 knockdown leading to an overactivation of RAS and AT1R signaling contributes to the hypertensive phenotype and reduced GFR in shGRK2 mice.

## Discussion

GRK2 is an important GRK isoform, as confirmed by the embryonic lethality of homozygous GRK2-deficient mice. This isoform is essential for cellular processes, such as cell signaling, cell cycle progression, migration and differentiation^10,15^. A mounting body of evidence indicates that GRK2 expression and activity are elevated in lymphocytes from hypertensive and heart failure patients^4,5^. These findings advanced the use of GRK2 inhibitors as a novel approach for treatment of hypertension and heart failure. Here, we studied mice with shRNA-mediated global shGRK2 knockdown to assess GRK2 role in renal function given the crucial role of the kidney in blood pressure homeostasis. We showed that GRK2 knockdown specifically affected kidney morphometry, reduced GFR and altered the ability of kidney to handle electrolytes. The reduction in GFR was accompanied by an increase in renal renin expression and activity in shGRK2 mice. This increase in renin activity was associated with a hypertensive phenotype that was sensitive to AT1R blocker, losartan. Overactivation of RAS was also evident in the renal cortex as an increase in ROS production and fibrotic glomerular sclerosis.

The components of the RAS (renin, angiotensinogen, ACE and AT1R) are synthesized locally in the kidney and the expression of each was higher in foetuses and newborn rats compared to adult rats^30^. This strongly indicates that the RAS is important for normal renal development. Histopathological abnormalities have been detected in mice with targeted homozygous deletions of the genes encoding renin, angiotensinogen, ACE, or AT1R^31^. Since GRK2 is crucial for the phosphorylation-dependent desensitization of AT1R receptors and termination of the signal^32^, knockdown of GRK2 is expected to pose detrimental abnormalities in renal development due to altered AT1R signaling. Our findings indicate that GRK2 knockdown reduced glomerulogenesis and kidney morphometry in postnatal and adulthood stages. AT1R is key for translating RAS actions in the kidney thus, reduced desensitization of AT1R due to GRK2 knockdown is expected to alter the overall effect of RAS on kidney development. It is noteworthy that the hypoplasia observed in the kidney was not evident in any other organ of the circulatory system such as lungs and heart that further emphasize the crucial role of GRK2 and AT1R signaling in nephrogenesis. Interestingly, Rivas *et al*., reported that hemizygous GRK2 mice exhibits altered pattern of vessel growth and maturation. This altered angiogenic response might also contribute to the impaired postnatal glomeruli maturation and reduction of glomeruli count in adult shGRK2 mice^33^. It is also worth noting that we excluded a compensatory role from other GRKs in shGRK2 mice since we did not detect any change in renal expression of other ubiquitously expressed GRKs (GRK3, 4, 5 and 6)^34,35^.

GFR measures the filtering capacity of the kidney and is determined by the filtration rate in each nephron and the number of filtering nephrons^36^. The reduction in mature glomeruli in postnatal periods of shGRK2 mice was maintained in adult mice. Moreover, we detected glomerulosclerosis and reduced GFR in adult shGRK2 mice. Mesangial cells are one of the main targets of Ang II in the renal cortex. Ang II via AT1R stimulates via mesangial cell growth and synthesis of the components of the extracellular matrix (collagen, fibronectin) and the type 1 inhibitor of plasminogen activator^37^. Moreover, along with its enzymatic properties, renin activates Ang II-independent signaling cascade via pro-renin receptors to triggers profibrotic and inflammatory injury in mesangial cells^24,38^. Thus, the increase in extracellular matrix protein production and glomerular sclerosis in adult shGRK2 nephrons could be attributed to overactivation of RAS and impaired AT1R desensitization.

Ang II is a powerful vasoconstrictor and it constricts both renal afferent and efferent arterioles as a result of AT1R activation. It increases afferent and efferent arteriolar resistances and reduces the glomerular filtration coefficient that results in decreases in GFR^39,40^. Thus, the loss of AT1R desensitization and overactivation of RAS following GRK2 knockdown can trigger a feedforward mechanism that maintains the reduction of GFR due to maintained of constriction afferent and efferent arterioles.

Renin is a rate-limiting enzyme in RAS and its expression and secretion are regulated at the juxta-glomerular apparatus by renal blood flow detected by macula densa^41,42^. Low renal blood flow increases renin secretion, stimulating Ang II and aldosterone production with a resulting increase in blood pressure and renal sodium retention^6,41,43^. Our findings indicate that the renal renin expression and plasma concentration are elevated in shGRK2 mice. We also detected a decrease in urinary Na^+^ concentration consistent with enhanced tubular reabsorption, an increase in serum K^+^ levels and elevated blood pressure in shGRK2 mice. These findings suggest that reduction in GFR in shGRK2 is sensed by macula densa to cause juxta-glomerular hypertrophy and increase the expression of renin to produce Ang II. Thus, the reduced blood flow in shGRK2 triggers a positive feedforward mechanism that increases Ang II production and sodium reabsorption leading to a hypertensive phenotype in adult shGRK2 mice and further reduction in GFR by constricting renal arterioles. The reduction in renal blood flow will be expected to reduce renal excretion of K^+^ and hyperkalemia that we detect in shGRK2 mice.

Accumulating evidence suggests that Ang II enhanced the production of reactive oxygen species (ROS) such as the superoxide anion and hydrogen peroxide through activation of NAD(P)H oxidase and increasing mitochondrial ROS formation^44^. ROS alters renal cells signaling, survival, and expression of inflammatory and extracellular matrix proteins. ROS production could be detrimental to the kidney depending on locally-generated concentrations and the counterbalance by antioxidant pathway^22,23,44^. Recently, activation of prorenin receptors by renin or prorenin was shown to induce fibrosis in cortical collecting duct cells via upregulation of collagen I and fibronectin that was dependent on the generation of ROS^24^. Our findings indicate that GRK2 knockdown was accompanied by an increase in superoxide and hydrogen peroxide production in renal cortices of mice. The increase in ROS production in shGRK2 kidneys could be attributed to overactivation of RAS and/or enhanced renal renin expression that can contribute to the sclerotic damage and renal injury. It is worth noting that we did not detect any changes in AT1R density or affinity in shGRK2 mice compared to age-matched control. This finding indicates the kidney injury and hypertensive phenotype in shGRK2 is likely due to upregulation of components of RAS leading to augmented AT1R signaling via ROS production.

Actions of the RAS in a variety of target organs have the potential to promote hypertension and end-organ damage including the kidneys. However, AT1R in the kidney was found to be primarily responsible for Ang II-mediated hypertension^5^. Indeed, following AT1 receptors deletion from the kidney, extrarenal AT1R was not sufficient to induce hypertension or cardiac hypertrophy^5^. These findings supported the crucial role of renal AT1R signaling in the pathophysiological changes leading to hypertension and other cardiovascular complications. Our findings further emphasize the crucial role of altered AT1R signaling in the kidney injury and the hypertensive phenotype of shGRK2 mice. Four-week treatment with AT1R blocker, losartan, completely reversed hypertension and partially corrected GFR in shGRK2 mice. Together, our results indicate the loss of AT1R desensitization following GRK2 knockdown leads to unopposed renal AT1R signaling that result in impaired renal filtration, electrolyte imbalance and hypertension in shGKR2 mice. However, we can not rule out the fact that altered AT1R signaling in tissues other than the kidney such as the heart and peripheral vasculature can contribute indirectly to the changes in renal blood flow and renal function.

It is worth noting that Avendaño and colleagues detected an enhanced vasoconstrictor responses to phenylephrine and endothelin 1 but not Ang II in aortic segments of hemizygous GRK2^+/−^ mice^45^. It is possible that the two knockdown approaches employed in our study and Avendaño *et al*. study will likely result in different GRK2 knockdown efficiency and compensatory regulation by other GRK isoforms. Different knockdown efficiency between GRK2^+/−^ and shGRK2 mice could be associated with variations in the level of AT1R desensitization that would be reflected at the physiological level. Moreover, kidney is a specialized organ that regulate blood pressure at multiple levels when compared to conduit and resistance vessels.

Our results indicate that although the use of GRK2 inhibitors for treatment of heart failure is promising; the benefits must be outweighed against their potentially deleterious effects on the kidney function. However, it is crucial to make the distinction between the genetic and the pharmacological approaches of GRK2 knockdown. Although genetic knockdown provides a selective tool, it can mask the relative importance of the kinase function versus the adaptor function of GRK2 in the context of kidney development and function. Thus, future studies using a selective GRK2 inhibitor are essential to validate our findings in the knockdown model.

In summary, we show that GRK2 downregulation can affect the glomerular development and count leading to reduced filtration and electrolyte imbalance. We also provided evidence that GRK2 knockdown was accompanied by overactivation RAS and enhanced AT1R-mediated ROS production that can further contribute to the glomerular injury, renal damage and hypertension. Our findings provided insights on the possible drawbacks for therapeutic inhibition of GRK2 at the level of kidney function that need to be considered prior to clinical application.

## Materials and Methods

### Animals

The GRK2 hemizygous (shGRK2) mice were generated on the C57Bl/6 genetic background as previously described^19^. Animals were housed in cages of two or more animals and were maintained on a 12 h light/12 h dark cycle at 24 °C. Mice received food and water *ad libitum*. All the experimental protocols and animal procedures were approved by the Animal Care Committee of the University of Ottawa (protocol #CMM 2519) and the University of Western Ontario (protocol #2009-081) and conducted in accordance with the guidelines and regulations of the Canadian Council on Animal Care.

### Morphometric Characterization of shGRK2 mice organ weights

Three sets of animals were sacrificed by exsanguination at 1.5, 3 and 6 months of age after recording total body weight for each animal. Hearts, kidneys, lungs and livers were collected, stripped of peritoneal fat and connective tissue and weights were obtained and recorded relative to body weight. Tibia length was measured and quantification of kidneys mass relative to tibia length was performed in a paired manner.

### Serum and urine analysis

Blood was collected from 1, 3 and 6-month-old shGRK2 and C57Bl/6 through cardiac puncture of mice under terminal anesthesia (ketamine/xylazine 100/10 mg/kg). Blood samples were centrifuged at 12,000 rpm for 10 minutes, serum was collected, and stored immediately at −80 °C for biochemical analysis. Urine samples from conscious, restrained mice were collected in the morning of the experiment. Biochemical analyses of serum and urine samples was performed by blinded technician at Mount Sinai Hospital Laboratory (Toronto, Ontario, Canada).

### Measurement of FITC-inulin clearance

The glomerular filtration rate (GFR) was measured in conscious mice by assessing plasma kinetics of fluorescein isothiocyanate (FITC) labeled inulin (Sigma Aldrich, Saint Louis, MO, USA). Briefly, 5% (w/v) FITC-inulin dissolved in 0.9% (w/v) saline was dialyzed overnight and sterilized by filtration. Anesthetized mice received a 3.74 μl/g bolus in the tail vein and blood samples from mice were collected into heparinized capillary tubes at 3, 7, 10, 15, 35, 55, and 75 minutes after injection then centrifuged for 10 minutes at 10,000 rpm. Samples were buffered in 500 mmol/L HEPES (pH 7.4), and plasma fluorescence was measured (Excitation 488 nm/Emission 538 nm). A two-compartment clearance model was used to calculate GFR as previously described^46^.

### RNA extraction and quantitative Real-Time Polymerase Chain Reaction

Kidneys were obtained and snap frozen after 6-month-old shGRK2 and C57Bl/6 were sacrificed by exsanguination. A total of 30 mg tissue was pulverized and homogenized using QIAshredder columns (Qiagen, Toronto, ON, Canada). Total RNA was obtained by using the RNeasy Minikit (Qiagen, Valencia, MD, USA) according manufacturer’s instructions. To minimize genomic contamination, an on-column DNase digestion step was included in the protocol. Two micrograms of total RNA were used to obtain cDNA using a High capacity cDNA reverse transcription kit (Applied Biosystems, Carlsbad, CA, USA). Quantitative Real -Time Polymerase Chain Reaction (qPCR) was employed to quantify the expression levels of genes of interest. TaqMan® Universal PCR Master Mix (Applied Biosystems, Foster City, CA, USA) was used. Six serial dilutions (1:10) of each target and reference genes served as a standard curve and was assayed together with the corresponding unknown samples on each plate. Reactions were performed in triplicate and delta-delta Ct (ΔΔCt) method was employed to determine the fold difference (2^−ΔΔCt^)^47^.

### Plasma Renin Analysis

Blood was collected in heparinized tubes from 6-month-old shGRK2 and C57Bl/6 through cardiac puncture under terminal anesthesia. Plasma were collected after configuration of blood samples at 12,000 rpm for 10 minutes. Plasma renin was measured by an enzyme-linked immunosorbent ELISA assay (Mouse Ren1-ELISA Kit, Thermo Fisher, Waltham, MA, USA) following manufacturer’s instructions. The optical density at 450 nm was determined for each sample in triplicate using an SynergyNeo2 multimode plate reader (Biotek, Winooski, VT, USA) and renin concentration was calculated from the standard curve.

### Western blotting

Snap frozen kidneys were lysed in 1.5 ml ice-cold lysis buffer (50 mM Tris, pH 8.0, 150 mM NaCl, and 1% Triton X-100) containing protease inhibitors (1 mM AEBSF, 10 μg/ml leupeptin, and 2.5 μg/ml aprotinin) and phosphatase inhibitors (10 mM NaF and 500 μM Na_3_VO_4_) and centrifuged at 15,000 rpm at 4 °C for 15 min. The supernatant was collected and total protein levels were quantified using Bradford Protein Assay (Bio-Rad). Homogenates were diluted in a mix of lysis buffer and β-mercaptoethanol containing 3x loading buffer and boiled for 10 min at 95 °C. Aliquots containing 25 μg total proteins were resolved by electrophoresis on a 7.5% SDS-polyacrylamide gel SDS-PAGE and transferred onto nitrocellulose membranes. Blots were blocked in Tris-buffered saline, pH 7.6 containing 0.05% of Tween 20 (TBST) and 5% non-fat dry milk for 2 hours at room temperature and then incubated overnight at 4 °C with Rabbit anti-GRK3, -GAPDH and -actin (Santa-Cruz biotechnology, Dallas, TX, USA) and mouse anti-renin (abcam Cambridge, MA, USA), -GRK2 (Sigma Aldrich), -GRK4, -GRK5 and -GRK6 (Santa-Cruz biotechnology) diluted 1:1000 in TBST containing 1% non-fat dry milk. Immunodetection was performed by incubating with secondary antibodies (anti-rabbit or -mouse, Thermo Fisher) diluted 1:5000 in TBST containing 1% of non-fat dry milk for 1 hour. Membranes were washed in TBS and then bands were detected and quantified using BioRad chemiluminescence system. Reagents used for western blotting were purchased from Bio-Rad Laboratories (Mississauga, ON, Canada). Full-length blots are provided in supplementary material file.

### Measurement of kidney superoxide and hydrogen peroxide

Renal content of superoxide (O_2_^−^) and hydrogen peroxide (H_2_O_2_) as indicators of oxidative stress were measured in kidney cortex lysates by high-performance liquid chromatography (HPLC) as described previously^48^. Briefly, fresh renal cortex pieces (20–30 mg) were incubated with 50uM dihydroethidium (DHE) (Sigma Aldrich) at 37 °C for 1 hour, homogenized and oxidation products were precipitated and resuspended in acetonitrile, and finally separated by HPLC into DHE, 2-hydroxydihydroethidium (2OH-Eth) and ethidium (Eth). Levels of oxidation products, superoxide-dependent 2OH-Eth and H_2_O_2_-dependent Eth, were normalized to DHE.

### Kidney histology

Mice were anesthetized with isoflurane and sacrificed by exsanguination and then perfused with phosphate buffered saline (PBS). kidneys were then excised, dissected and immediately fixed in 4% paraformaldehyde (PFA, Sigma Aldrich). Paraffin-embedded kidney sections were obtained and stained with Periodic-Acid Schiff (PAS, Sigma Aldrich) or hematoxylin-eosin (H&E, Sigma Aldrich). All sectioning, paraffin embedding and staining were performed by the University of Ottawa’s pathology department. Kidney sections were viewed under light-microscopy at either 40x, 100x or 400x magnification (Axioskop 2 Imager A1, Zeiss, Germany). Representative glomerular profiles for each group (n = 4–5 mice/group) were analyzed in a blinded manner by a trained pathologist. Glomerular sclerosis was scored (0–3) based on mesangial matrix area. For assessment of renal development, glomeruli were counted in 8–10 low magnification images both H&E and PAS stained kidney sections from P7 (n = 4 per group). Assessment of glomerular number of 6-month-old mice was performed using the point selection tool with auto measure of Image J software (Bethesda, MD, USA). The mean glomerular number per unit area of tissue section (mm^2^) in adult animals was calculated from a total number of 160 microscopic fields for H&E -stained sections.

### AT1R binding experiments

Saturation binding assays using mouse kidney were performed to determine the affinity (K_d_) and the density (B_max_) of renal AT1R for each genotype. ^125^I-AngII (specific radioactivity ~1000 Ci/mmol) was prepared with Iodo-GEN® (Perbio Science, Erembodegem, Belgium) as reported previously^49^. 6-month-old shGRK2 and age-matched C57Bl/6 mice were euthanized by exsanguination. The kidney was then rapidly dissected and kept at −80 °C until homogenization. Isolated kidneys were homogenized using a Polytron Generator F125-7 mm stator (Thermofisher) followed by an electronic 10 ml Teflon homogenizer (Kinematica, Inc., Bohemia, NY, USA) in 5 ml of ice-cold PBS at pH 7.4 with protease inhibitors. The preparation was then centrifuged at 2,000 rpm for 15 minutes at 4 °C. The supernatants were then centrifuged at 27,000 g (S150AT-0162 rotor; Sorvall Discovery M150) for 30 minutes at 4 °C, the supernatant was discarded, and the pellets were used immediately. The pellets were resuspended in washing buffer (25 mM Tris-HCl, pH 7.4, 100 mM NaCl, 5 mM MgCl_2_), protein concentrations were determined then the preparation was diluted in binding buffer (25 mM Tris-HCl, pH 7.4, 100 mM NaCl, 5 mM MgCl_2_, 0.1% bovine serum albumin, 0.01% bacitracin). Saturation binding experiments were done by incubating membrane preparation (50 μg of protein) for 1 h at room temperature with increasing concentrations (0.25 nM–8 nM) of ^125^I-AngII in a final volume of 500 μl. Nonspecific binding was determined in the presence of 1 μM unlabeled Ang II. Bound radioactivity was separated from free ligand by filtration through GF/C filters presoaked for at least 1 h in binding buffer. Receptor-bound radioactivity was evaluated by γ counting. Results are presented as means ± S.E.M. Binding data (B_max_ and K_d_) were analyzed with GraphPad prism using a one-site binding hyperbola nonlinear regression analysis.

### Losartan treatment experimental design

Two sets of shGRK2 and age-matched C57Bl/6 mice were aged to 6 months and then each set was divided into two groups. One group from each set either received 100 mg/kg/day Losartan (Sigma Aldrich) in drinking water for 4 weeks or plain drinking water. Water intake was measured daily and was approximately 25 ml/week/mouse. No differences in the water intake or urine output was detected between groups. Systolic and diastolic blood pressures were blindly measured before treatment start, weekly during the 4-week period of treatment and 3 weeks after washout ended using the non-invasive CODA tail-cuff blood pressure system (Kent Scientific, Torrington, CT, USA). To eliminate the influence of circadian rhythm, blood pressure was measured at the same time of day. Mean arterial blood pressure (MAP) was calculated form the following equation MAP = diastolic + [(systolic-diastolic)/3]. At the end of the 4-week treatments mice, GFR was measure using FITC-inulin clearance described earlier.

### Statistical analysis

Statistical differences between groups were checked using GraphPad Prism software version 7. Data were analyzed with either unpaired Student’s t test or two-way ANOVA followed by Fisher’s LSD comparison as appropriate. A two-tailed p < 0.05 was considered significant.

### Data availability

All data generated or analyzed during this study are included in this published article.

## Electronic supplementary material

Supplementary data
