# Supplementary material for: GRK2 knockdown in mice exacerbates kidney injury and alters renal mechanisms of blood pressure regulation
Source: Sci Rep. 2018 Jul 30;8:11415. doi: 10.1038/s41598-018-29876-8 (PMC6065385; doi:10.1038/s41598-018-29876-8)
Supplement: Supplementary file 1 — Supplementary data [file 41598_2018_29876_MOESM1_ESM.docx]

**GRK2 knockdown in mice exacerbates kidney injury and
alters renal mechanisms of blood pressure regulation**

**Elena Tutunea-Fatan^1,2,#^, Khaled S. Abd-Elrahman^3,5,#^, Jean-Francois Thibodeau^3,4^, Chet E. Holterman^4^, Brian J. Holleran^6^, Richard Leduc^6^, Christopher R.J. Kennedy^3,4^, Robert Gros^1,2^, and Stephen S. G. Ferguson^3,*^**

^1^Vascular Biology Group, Robarts Research Institute, ^2^Department of Physiology and Pharmacology, University of Western Ontario, London, Ontario N6A 5K8, Canada

^3^University of Ottawa Brain and Mind Research Institute and Department of Cellular and Molecular Medicine, University of Ottawa, Ottawa, Ontario K1H 8M5, Canada

^4^Kidney Research Center, Ottawa Hospital Research Institute, Ottawa, Ontario, K1H 8M5, Canada

^5^Department of Pharmacology and Toxicology, Faculty of Pharmacy, University of Alexandria, Alexandria, 21521, Egypt.

^6^Department of Pharmacology-Physiology, Faculty of Medicine and Health Sciences, Université de Sherbrooke, Sherbrooke, Quebec, Canada, J1H 5N4.

**#** these authors contributed equally to this work

*****Address correspondence to:

Dr. Stephen S. G. Ferguson

Department of Cellular and Molecular Medicine, University of Ottawa,

451 Smyth Dr. Ottawa, Ontario, Canada, K1H 8M5. Tel: (613) 562 5800 Ext 8889. sferguso@uottawa.ca

## Supplementary figure 1


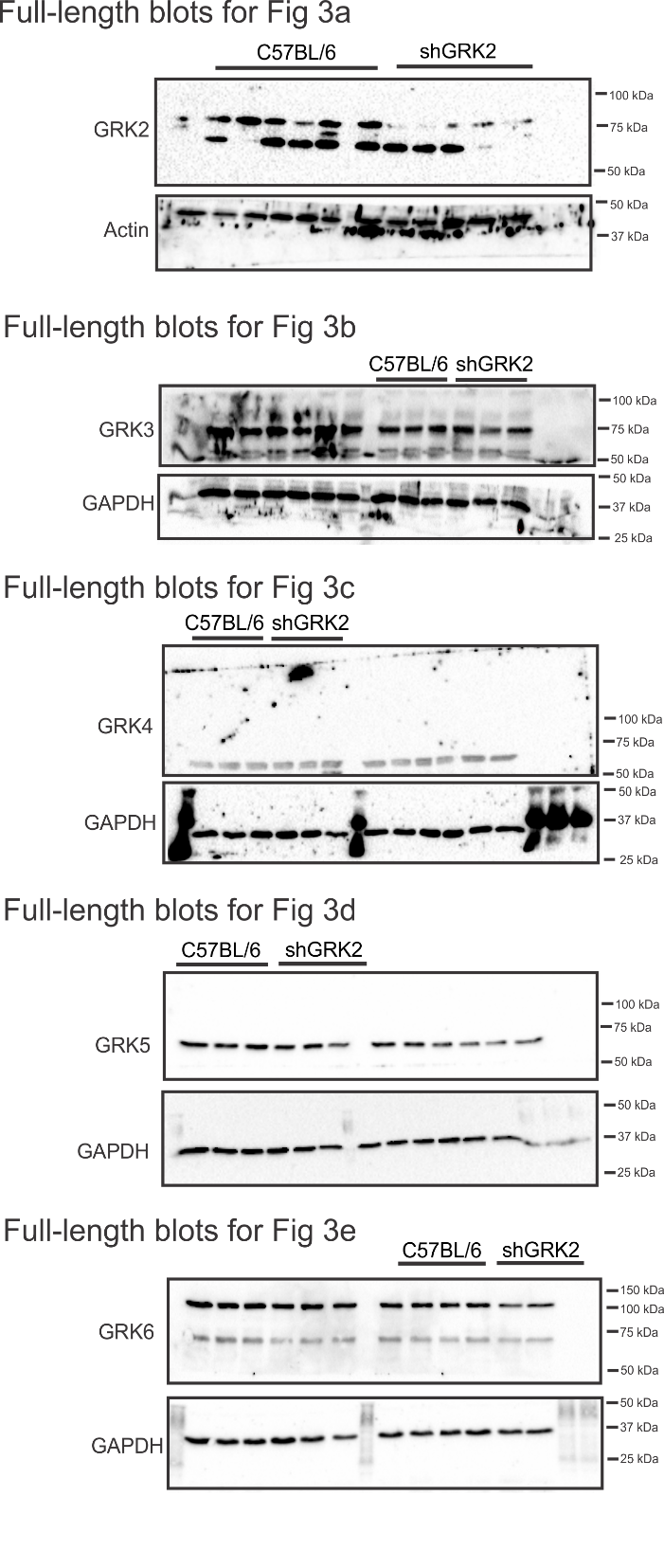


**Supplementary figure 1:** Full length blots corresponding to figure 3

## Supplementary figure 2

**
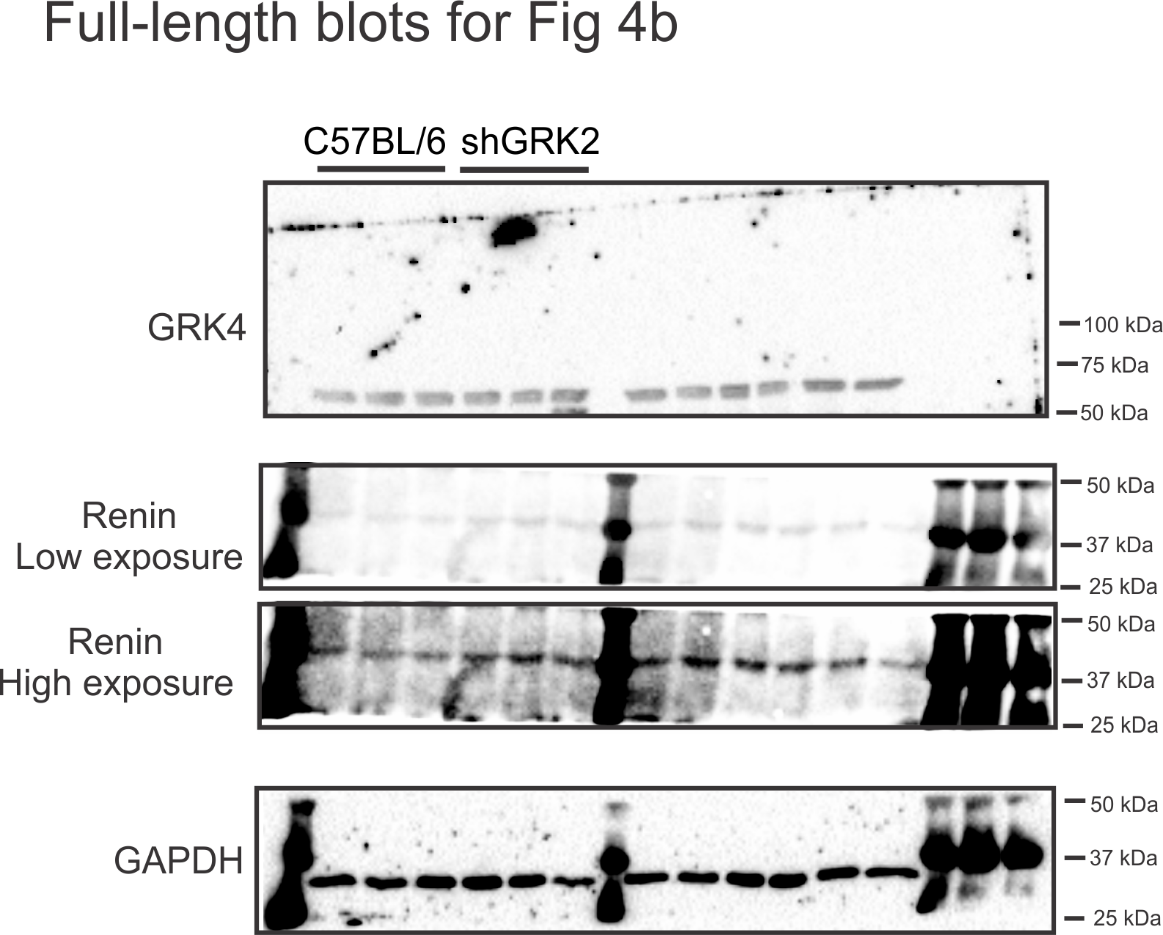
**

**Supplementary figure 2:** Full length blots corresponding to figure 4b. The full-length blot was used to generate figure 3a and 4b hence the same loading control (GAPDH) was employed in both figures. Two exposure settings are displayed for renin blots. The high exposure renin blot corresponds to figure 4b in the main text. The bottom part of the blot was probed for renin then re-probed for GAPDH.
